# *In silico* analysis of PFN1 related to amyotrophic lateral sclerosis

**DOI:** 10.1371/journal.pone.0215723

**Published:** 2019-06-19

**Authors:** Gabriel Rodrigues Coutinho Pereira, Giovanni Henrique Almeida Silva Tellini, Joelma Freire De Mesquita

**Affiliations:** Department of Genetics and Molecular Biology, Bioinformatics and Computational Biology Laboratory, Federal University of the State of Rio de Janeiro (UNIRIO), Rio de Janeiro, Rio de Janeiro, Brazil; King's College London, UNITED KINGDOM

## Abstract

Profilin 1 (PFN1) protein plays key roles in neuronal growth and differentiation, membrane trafficking, and regulation of the actin cytoskeleton. Four natural variants of PFN1 were described as related to ALS, the most common adult-onset motor neuron disorder. However, the pathological mechanism of PFN1 in ALS is not yet completely understood. The goal of this work is to thoroughly analyze the effects of the ALS-related mutations on PFN1 structure and function using computational simulations. Here, PhD-SNP, PMUT, PolyPhen-2, SIFT, SNAP, SNPS&GO, SAAP, nsSNPAnalyzer, SNPeffect4.0 and I-Mutant2.0 were used to predict the functional and stability effects of PFN1 mutations. ConSurf was used for the evolutionary conservation analysis, and GROMACS was used to perform the MD simulations. The mutations C71G, M114T, and G118V, but not E117G, were predicted as deleterious by most of the functional prediction algorithms that were used. The stability prediction indicated that the ALS-related mutations could destabilize PFN1. The ConSurf analysis indicated that the mutation C71G, M114T, E117G, and G118V occur in highly conserved positions. The MD results indicated that the studied mutations could affect the PFN1 flexibility at the actin and PLP-binding domains, and consequently, their intermolecular interactions. It may be therefore related to the functional impairment of PFN1 upon C71G, M114T, E117G and G118V mutations, and their involvement in ALS development. We also developed a database, SNPMOL (http://www.snpmol.org/), containing the results presented on this paper for biologists and clinicians to exploit PFN1 and its natural variants.

## Introduction

Amyotrophic lateral sclerosis (ALS) is a neurodegenerative disease that progressively affects the upper and lower motor neurons, leading to muscular atrophy and paralysis due to neuron injury and death [1]. ALS is the most common adult-onset motor neuron disorder [2] with an estimated economic burden of over one billion dollars a year in the United States only [3]. Due to the lack of effective treatments, ALS leads to death within 2 to 5 years after the diagnosis, usually due to respiratory paralysis [4]. Most ALS cases are sporadic (sALS); however, 5–10% of the ALS cases are familial (fALS) and related to genetic causes [5].

Four non-synonymous single nucleotide variants (nsSNVs) in the *PFN1* gene were described as being involved with fALS development [6,7]. Interestingly, these mutations were also found in sporadic cases of ALS [8]. The *PFN1* gene encodes profilin 1 (PFN1), a 140-residues ubiquitously expressed [9] cytosolic protein [10] that plays key roles in the regulation of actin cytoskeleton [11].

PFN1 is crucial for monomeric actin conversion into filamentous actin, as it sequestrates cytosolic actin monomers and catalyzes the assembly of monomers into filamentous-actin [9]. PFN1 also interact with poly-L-proline (PLP) sequences and major proline-rich protein families, such as vasodilator-stimulated phosphoproteins (VASP), which participates of the nucleation and elongation of actin filaments. PFN1 interaction with these cytoskeleton regulators is an important generator of actin-based structures [12]. Previous studies have shown that PFN1 is also an important regulator of cell motility events, including migration and invasion of breast cancer and vascular endothelial cells. Furthermore, disrupted PFN1 interactions, as well as reduced PFN1 expression have been shown to cause impaired capillary morphogenesis and defects in neurite development [13].

Moreover, PFN1 is involved in many cellular processes [11] through the interaction with diverse binding partners [14], including structural proteins in neurons, growth factors [9], ribonuclear particles [15] and proteins involved in signaling cascades [9]. PFN1 also plays important roles in membrane trafficking [16], RNA processing and transcription [9], GTPase signaling [17], and neuronal growth and differentiation [16]. In neurons, PFN1 is essential for neuronal development, formation and maintenance of the neuronal cytoskeleton, synaptic formation and activities, as well as growth of dendrites and axons [8].

ALS-related mutations in PFN1 are known to cause cytoskeletal disruption in neurons [10], resulting in axonal dysfunction and retraction. This leads to synaptic failure with consequent denervation of post-synaptic motor neurons [18]. Cytoskeletal defects plays a major role in motor neuron diseases and contributes importantly to ALS pathogenesis [19]. It is also known that PFN1 mutations cause proteostasis disturbances [14], which are evidenced by the presence of biological markers, such as formation of cytoplasmic protein inclusions [10] and accumulation of ubiquitin and p62 [20]. PFN1 mutations are known to destabilize PFN1 resulting in structural perturbations that lead to protein aggregation [17]. Protein misfolding and aggregation result in proteostasis network disturbance, which is believed to contribute to early events in ALS pathogenesis [21]. Thus, studying the PFN1 missense mutations may contribute to a better understanding of the ALS pathophysiology.

Next-generation sequencing experiments reveal millions of novel SNVs [22]. However, the experimental characterization of their effects is extremely expensive, time-consuming and difficult [23]. The computational simulations, also known as *in silico* analysis, allows the prediction of SNV effects in a faster, cheaper and efficient way [4]. The computational approach is then beneficial in prioritizing the most probable disease-related mutations [23] to be narrowly examined with wet-lab experiments [4]. Moreover, already known disease-related mutations can also be studied *in silico* to identify pharmacological targets for relevant treatments and to gain insight into their molecular mechanisms of pathology [23]. In this scenario, the computational simulations have become an important ally of the experimental methods [4] and an essential approach for the study of SNVs [22,23].

Optimal protein-drug binding is crucial to achieving the desired therapeutic effects, as well as to minimizing associated side effects and toxicity of drugs. Protein-drug interactions are determined by local biochemical and structural features of drug-binding cavities [24]. Residues outside drug-binding cavities can also have long-range effects on these sites and, consequently, influence protein-drug binding [25]. Thus, key amino-acid residues in proteins are essential for maintaining the structural properties of binding sites and for the formation of non-covalent interactions with drug molecules [24]. In this sense, nsSNVs affecting key protein residues can impact drug binding-sites, resulting in alterations in drug binding affinity and selectivity [26].

In this work, we applied computational simulations, following the methodology previously established by our group [4,27,28], to the study of PFN1 nsSNVs, which were described as related to ALS development [6,7]. We aim at the characterization of the PFN1 nsSNVs and their effects on protein structure and function. Here, we applied ten functional and stability prediction algorithms, an evolutionary algorithm and molecular dynamics simulations to a thorough analysis of PFN1 nsSNVs. Our findings suggested that these nsSNVs could affect PFN1 flexibility, which could be therefore related to ALS development. We also developed an database containing the results presented in this paper for biologists and clinicians to exploit PFN1 and its natural variants.

Since these nsSNVs may influence drug selection, dosing, and adverse effects, understanding their effects on PFN1 structure and function may help the development of new drugs and personalized therapies for ALS [22].

## Materials and methods

### Sequence, structure and natural variants retrieval

The sequence and natural variants of PFN1 were retrieved from the UniProt database (UniProt ID: P07737) [7]. The structure of and the wild-type PFN1 was retrieved from the Protein Data Bank (PDB) database (PDB ID: 1PFL) [29].

### Functional and stability prediction analysis

The functional and stability effects of the PFN1 nsSNVs were predicted using the following algorithms: PhD-SNP [30], PMUT [31], PolyPhen-2 [32], SIFT [33], SNAP [34], SNPS&GO [35], SAAP [36], nsSNPAnalyzer [37], SNPeffect4.0 [22] and I-Mutant2.0 [38].

### Evolutionary conservation analysis

The evolutionary conservation analysis of PFN1 was performed using the ConSurf server, which determined the degree of evolutionary conservation of each amino-acid of PFN1 [39]. The following parameters were selected for this analysis: PDB ID: 1PFL; Chain identifier: A; homologous search algorithm: PSI-BLAST; number of iterations: 3; E-value cut-off: 0.0001; protein database: UniProt; reference sequence: closest; number of reference sequences selected: 150; maximum sequence identity: 95%; minimum identity for counterparts: 35%; alignment method: MAFFT-L-INS-i; calculation method: Bayesian; and evolutionary substitution model: best model (default).

### Molecular dynamics simulations

MD simulations of the wild-type PNF1 and its natural variants: C71G, M114T, E117G and G118V, were performed using the GROMACS 2018.2 package [40]. Mutator Plugin 1.3 [41], which is available in the Visual Molecular Dynamics (VMD) 1.9.1 software [42] was used to induce the C71G, M114T, E117G and G118V substitution on the experimentally determined structure of wild type PFN1 (PDB ID: 1PFL) [29].

Following the methodology previously established by our group [4], we selected the amber99SB-ILDN as the force field of the simulations. Amber99SB-ILDN is an improved version of the amber99SB force field [43], which is widely used in MD simulations of proteins [44]. The new side-chain torsion potentials of amber99SB-ILDN are clearly improved and do not cause undesirable side effects [43]. Amber99SB-ILDN proved to be a good choice for the MD simulation of proteins [44], since this force field accurately descript many protein structural and dynamical properties [45]. Amber99SB-ILDN is therefore recommended for the simulation of protein dynamics [43,44].

The structures were solvated using the TIP3P water model inside a dodecahedral box of dimensions 44 x 37 x 34 Å. The systems were neutralized by adding Na^+^ and Cl^−^ ions and minimized for 5000 steps using the steepest descent method.

After system minimization, three other steps were carried out in the MD simulations: NVT (constant number of particles, volume, and temperature), NPT (constant number of particles, pressure, and temperature) and production. The NVT ensemble was followed by the NPT ensemble at 1 atmosphere and temperature of 300 K for the duration of 100 ps [4]. Parrinello-Rahman was selected as the barostat and v-rescale was selected as the thermostat of the NVT and NPT ensembles.

The production simulations were performed in triplicates at 300 K for the duration of 100 ns for the wild-type PFN1 and its variants. The LINCS (linear constraint solver) algorithm was applied to constrain covalent bonds [46], and the electrostatic interactions were processed using the particle mesh Ewald (PME) method [47]. The time step of 0.002 ps was selected for the simulations and the MD trajectories were recorded every 10 ps [4].

Structural parameters of the wild-type PFN1 and its variants were accessed through the root-mean-square-deviation (RMSD), root-mean square-fluctuation (RMSF), radius of gyration (Rg), intramolecular hydrogen bonds (Hb) and B-factor analyses. These parameters were calculated separately for each triplicate trajectory. The means for each triplicate in the RMSD, RMSF, RG and intramolecular Hb analyses were calculated and plotted using the ggplot2 package in R software [48].

The following GROMACS distribution programs were used to perform the MD analyses: *gmx hbond*, *gmx rms*, *gmx rmsf*, *and gmx gyrate*.

### PFN1 database development

The results presented in this paper were compiled and stored on SNPMOL, an online database. The human-curated database of PFN1 was developed using JSmol, an HTML5-based equivalent of Jmol [49].

## Results and discussion

### Sequence, structure and natural variants retrieval

PFN1 is a 140-amino acid cytoskeletal protein that is coded by the *PFN1 gene* [7], which is located on chromosome 17p13.2 [50]. Four natural variants of PFN1 were described as related to the development of ALS type 18 [7] structure, i.e., PDB ID: 1PFL, experimentally determined by nuclear magnetic resonance (NMR) spectroscopy [9,29].

PFN1 protein has two important domains: an actin-binding domain and a poly-L-proline (PLP) binding domain [9], which are essential for PFN1 to perform its biological functions [9,12]. The actin-binding domain of PFN1 is located on its helix 3 and part of its strands 4, 5 and 6, whereas the PLP binding domain is lo’cated on the N and C terminal helices [9,15,16]. Moreover, the residue threonine 89 (T89) is an important site of PFN1, which is phosphorylated by PKA. The phosphorylation of T89 was predicted to potentially increase the PFN1 affinity for actin. This post-translational modification is believed to be a regulatory mechanism of PFN1-dependent actin polymerization processes. Moreover, several changes were observed by inducing the T89D mutation in PFN1, including detergent insolubility, protein aggregation and accelerated proteolysis, which suggested that the T89 residue is structurally important for PFN1 [51].

**Fig 1:**
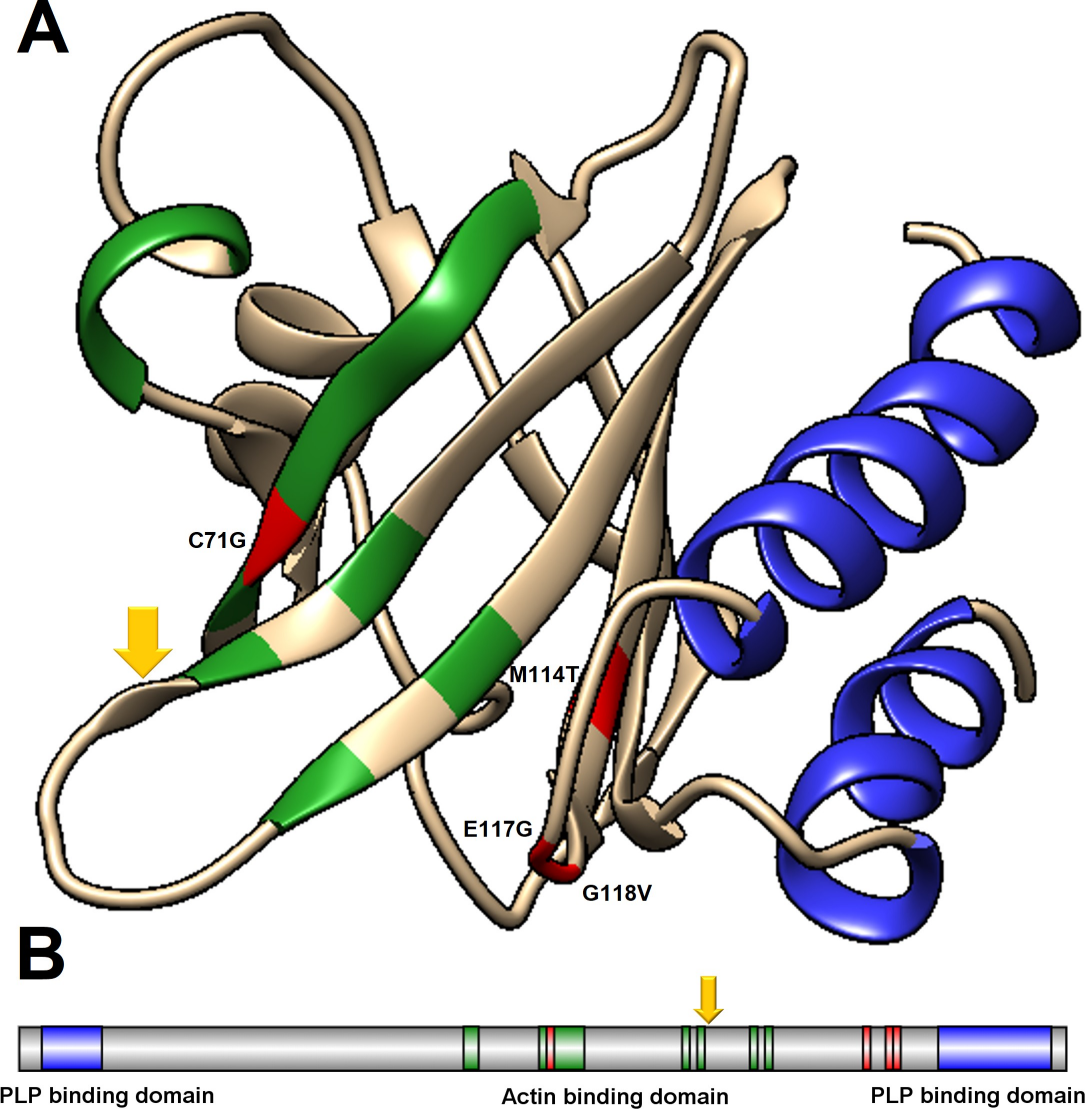
Tridimensional structure and schematic representation of PFN1. The PLP binding domain and the actin-binding domain of PFN1 are represented in blue and green, respectively. The mutation sites: C71, M114, E117 and G118, are represented in red. The dark yellow arrow shows the residue threonine 89. (A) Tridimensional structure of PFN1 (PDB ID: 1PFL). (B) Schematic representation of PFN1.

A schematic representation of PFN1 containing its natural variants and important domains are shown in Fig 1. As shown in Fig 1, all studied PFN1 nsSNVs lead to amino acid substitutions in regions that are spatially close to the actin binding and PLP binding domains of the protein. It is believed to be related to the impaired actin-binding ability and altered PLP-binding ability of the PFN1 ALS-related variants [9,16].

### Functional and stability prediction analysis

**Fig 2:**
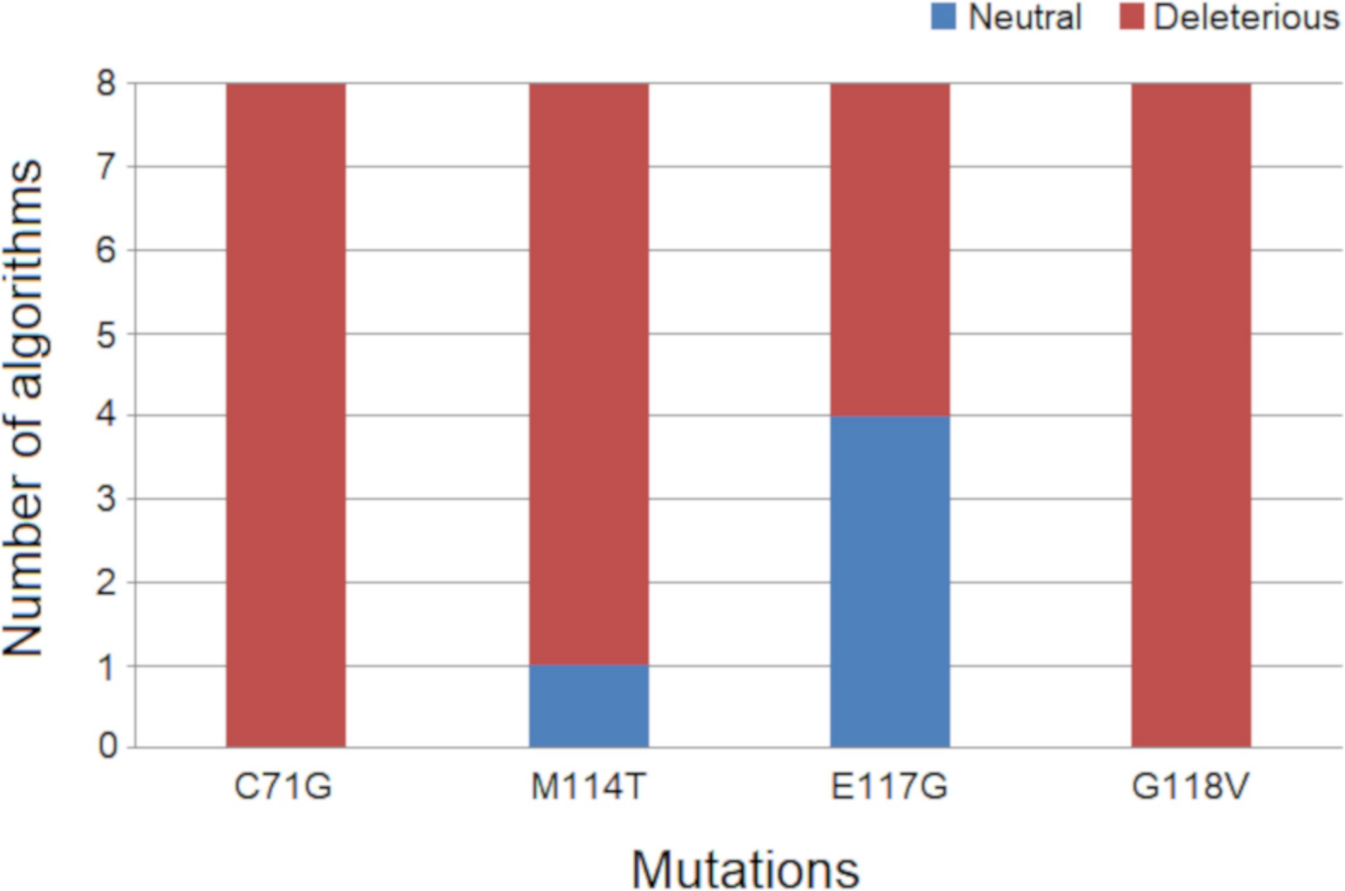
Functional prediction results of each PFN1 nsSNV. The four known nsSNVs of PFN1 were analyzed using eight different functional prediction algorithms. The bar plot indicates the number of neutral and deleterious predictions of each PFN1 nsSNV, according to the used algorithms. Blue bars indicate neutral predictions while red bars indicate the number of deleterious predictions.

The functional and structural consequences of nsSNVs at the protein level can be predicted using computational simulations [52]. The effects of amino acid substitutions on PFN1 function were analyzed using eight different algorithms. The mutations C71G and G118V were predicted as deleterious by the eight functional prediction algorithms that were used. The M114T mutation, in turn, was predicted as deleterious by seven of the eight algorithms, while the E117G mutation was predicted as deleterious by four of the eight algorithms (Fig 2).

In the test case we performed, the algorithms: SAAP, SIFT, SNAP, and SNPs&GO, showed the best accuracy amongst the used functional prediction algorithms. They were able to detect the known deleterious effects of the studied PFN1 mutations [7]. The PhD-SNP algorithm presented the worst accuracy in the test case we performed, as it was not able to detect the known deleterious effects of the M114T and E117G mutations [7] (Table 1).

**Table 1:** Functional prediction analysis of PFN1 natural variants.

	Functional prediction algorithms							
Natural variant	nsSNPAnalyzer	PhD-SNP	PolyPhen-2	PMut	SAAP	SIFT	SNAP	SNPs&GO
**C71G**	Deleterious	Deleterious	Deleterious	Deleterious	Deleterious	Deleterious	Deleterious	Deleterious
**M114T**	Deleterious	Neutral	Deleterious	Deleterious	Deleterious	Deleterious	Deleterious	Deleterious
**E117G**	Neutral	Neutral	Neutral	Neutral	Deleterious	Deleterious	Deleterious	Deleterious
**G118V**	Deleterious	Deleterious	Deleterious	Deleterious	Deleterious	Deleterious	Deleterious	Deleterious

Despite the high accuracy in detecting the known deleterious effects of C71G, M114T and G118V, the algorithms that were used showed low accuracy in predicting the known deleterious effect of the E117G variant of PFN1. These algorithms apply different strategies to make predictions [28]. Moreover, there is no established gold standard method to predict the functional effects of mutations [53]. Thus, it is important to combine the results of a variety of algorithms to determine the deleterious effects of mutations, as previously demonstrated by our group [4,28,54]. The test case we performed reaffirms the importance of the combined usage of algorithms when proceeding predictive functional analysis. The divergent results and the weaknesses of functional prediction algorithms evidence the need of improving such methods.

The effects of amino acid substitutions on PFN1 stability were further analyzed using the FoldX [55] and I-Mutant2.0 [38] algorithms. According to I-Mutant2.0 and FoldX, the mutations C71G, M114T and E117G decrease PFN1 stability. The mutation G118V, in turn, was predicted as destabilizing for FoldX and stabilizing for I-Mutant2.0. Recently, Boopathy *et al*. [16] showed that the ALS-related mutations: C71G, M114T, and G118V, but not E117G, destabilize PFN1 *in vitro* [9,16].

The divergent results presented in the stability prediction analysis may occur due to the different prediction strategies applied by I-Mutant2.0 and FoldX [22,38]. While FoldX is an algorithm trained in a database of engineered proteins [55], I-Mutant 2.0 uses information from a database of experimentally determined structures to predicted the effect of mutations on protein stability [38].

Lastly, the effects of amino acid substitutions on PFN1 aggregation tendency (TANGO), amyloid propensity (WALTZ), and chaperone binding tendency (LIMBO) were analyzed using the SNPeffect4.0 algorithm [22]. According to SNPeffect4.0, none of these mutations affect the PFN1 aggregation tendency, amyloid propensity, and chaperone binding tendency. Interestingly, the protein variants: C71G, M114T and G118V, are known to aggregate *in vitro* [19].

### Evolutionary conservation analysis

ConSurf is a bioinformatics tool that analyzes the evolutionary conservation of protein regions and calculates the conservation score of each amino acid based on statistical inference methods, machine learning, and multiple sequence alignments. The conservation scores are associated with a coloring scheme and projected on the protein’s surface. ConSurf is widely used to detect functional regions on proteins as important residues are usually conserved throughout evolution [39].

**Fig 3:**
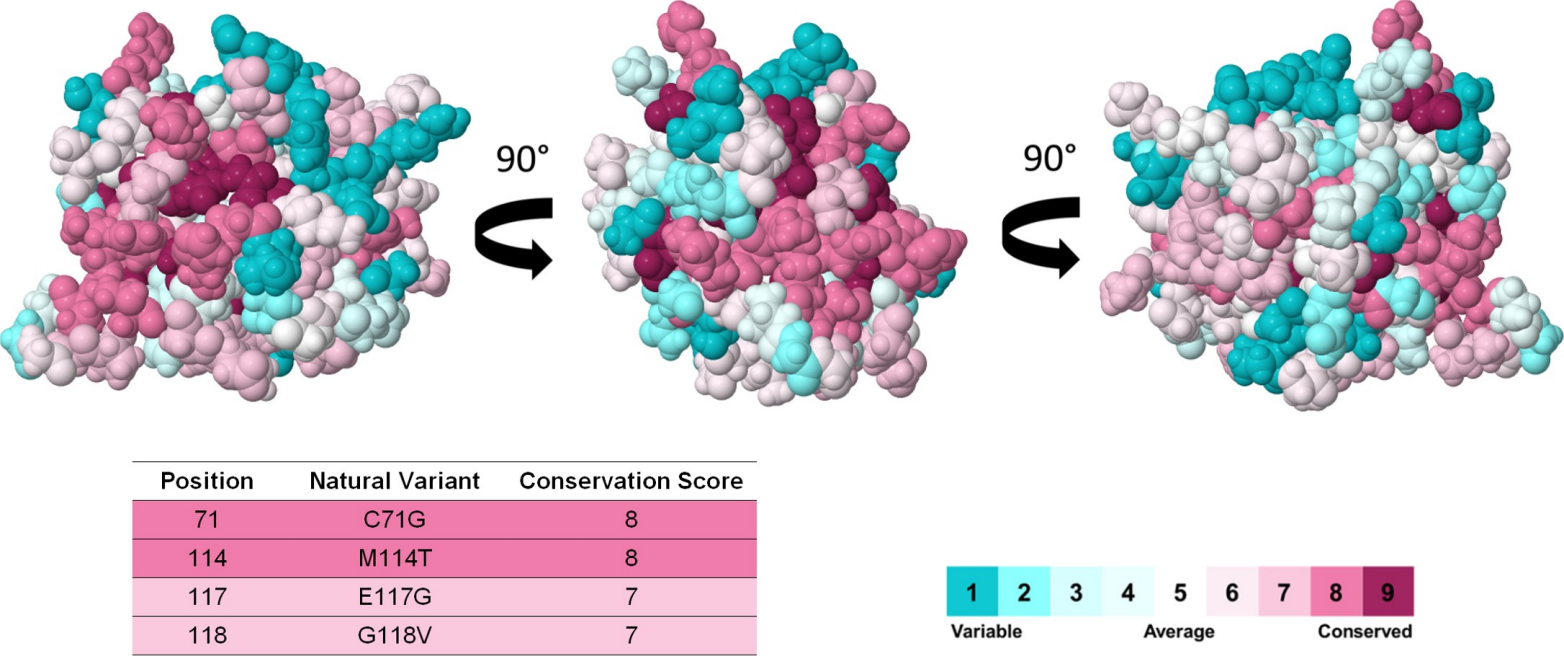
Evolutionary conservation analysis of PFN1. The PFN1 conservation profile shown in three different angles. Each PFN1 amino acid is represented as a space-filling model and colored according to its conservation score. The ConSurf coloring scheme is shown in the color-coding bar. According to ConSurf, the positions 71, 114 and 118 are highly conserved, while the position 117 is average conserved.

The evolutionary conservation score of each amino acid of PFN1 was calculated by ConSurf (Fig 3). Highly conserved positions are colored maroon, average conserved positions are colored white, and variable positions are colored turquoise [39]. According to ConSurf, all PFN1 mutations occur in conserved positions, which indicate that these variants probably affect important PFN1 sites. It might explain the association of these mutations with ALS development. Moreover, PFN1 has two major areas composed of structural conserved amino acids, which correspond to the actin binding domain and adjacent residues, as well as the PLP-binding domain. These regions are crucial to PFN1 performs its biological function [14], which probably contributed to their structural conservation throughout the evolution [39].

In addition to showing the conservation scores of PFN1 mutated sites, the ConSurf analysis also provided an interesting graphical representation in which the conservation scores for amino acid of PFN1 is plotted on its three-dimensional protein structure, highlighting its conserved regions and structural proximities.

### Molecular dynamics simulations

MD is an *in silico* method of solving Newtonian equations of motions for a given set of atoms [56]. This method aims to reproduce the real behavior of molecules, such as proteins, in their environment. Unlike the static pictures obtained from methods such as X-ray crystallography [4], the molecular trajectories generated by MD simulations provide detailed information on changes in protein conformation and fluctuation. This information can be used to assess structural parameters of proteins, such as flexibility and stability [57]. As changes in protein flexibility and stability may lead to the development of pathologies [52,58,59], the impact of mutations on protein structure and function can be understood using MD simulations (Vinay Kumar *et al*., 2014).

To further analyze the effects of PFN1 nsSNVs we carried out MD simulations of the wild-type PFN1 and its four natural variants [40] using the GROMACS 5.0.7 package [40]. The NMR structure of PFN1 (PDB ID: 1PFL) was used as the wild type structure. The tridimensional structures of the C71G, M114T, E117G and G118V variants were generated by inducing the respective amino acid substitutions on the wild type PFN1 using the VMD software (Version 1.9.1) [42]. The MD simulations of the wild-type PFN1 and its natural variants were carried out for 100ns. The generated trajectories were evaluated according to their RMSD, RMSF, RG, intramolecular Hb and B-factor characteristics.

**Fig 4:**
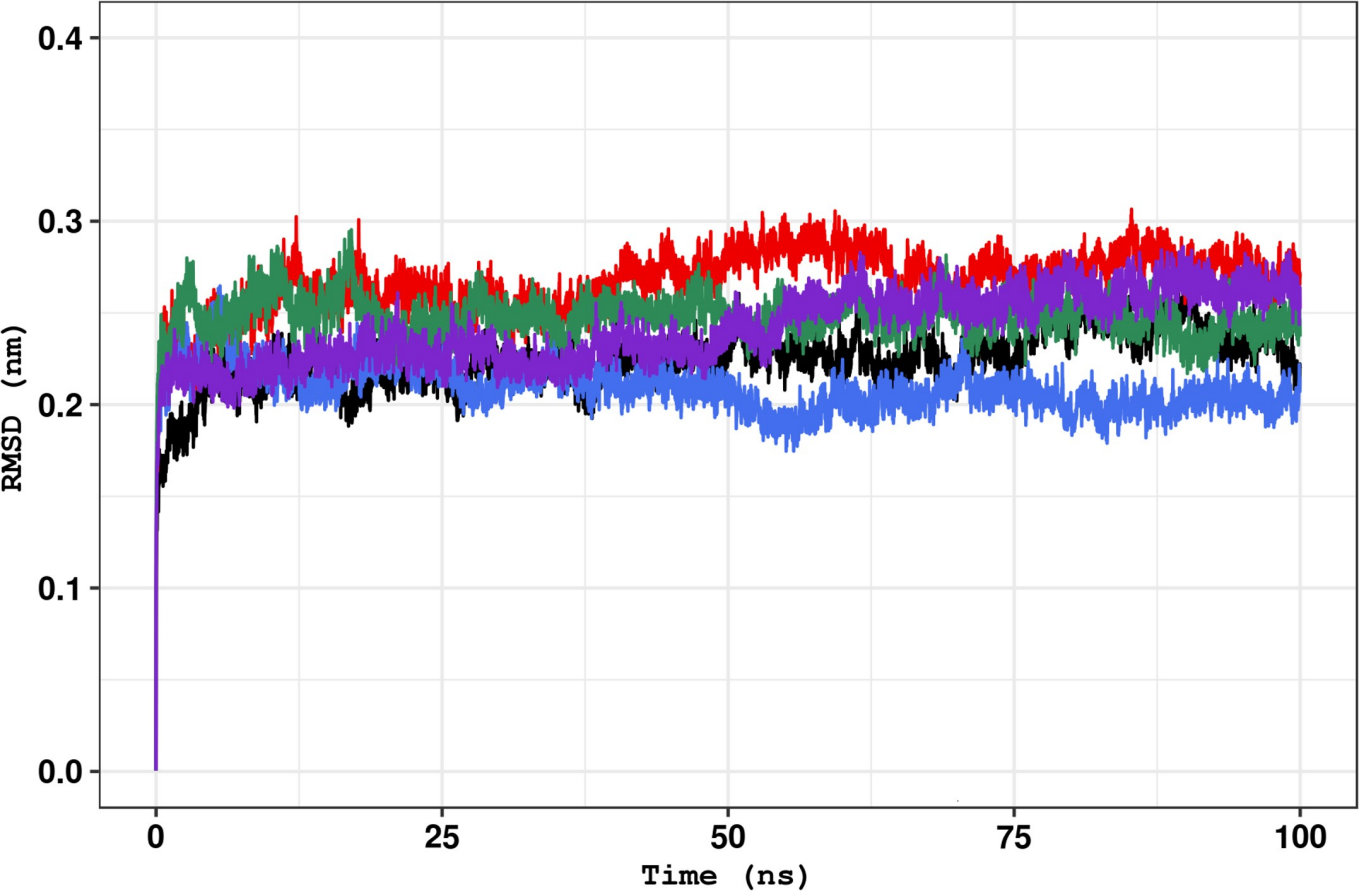
Backbone RMSD of the wild-type PFN1 and its natural variants. The RMSD for the backbone atoms of the wild-type structure and variants at 300K shown as a function of time. The wild type is represented in black,variant C71G is represented in red, variant M114T is represented in blue, variant E117G is represented in green, and variant G118V is represented in purple.

RMSD is a useful parameter to analyze the structure motions over time and to determine its spatial convergence throughout the simulation [4,56,60]. As shown in Fig 4, the average RMSD values of the C71G (0.1875±0.02nm), M114T (0.2091±0.02nm), E117G (0.2480±0.02nm), and G118V (0.2415±0.3nm) variants are similar to the wild-type PFN1 (0.2248±0.04nm). It indicates that the PFN1 variants diverge from the initial position as much as the wild-type PFN1. Moreover, the establishment of a plateau in the RMSD values, observed in all simulations (Fig 4), suggests that the structures fluctuate around an average stable conformation, thus making sense to assess its local fluctuations [56,60]. The E117G simulation reached a plateau of RMSD values first (around 25ns), followed by the wild-type (around 40ns), M114T(around 60ns), G118V(around 65ns), and C71Gsimulations (around 70ns), respectively.

**Fig 5:**
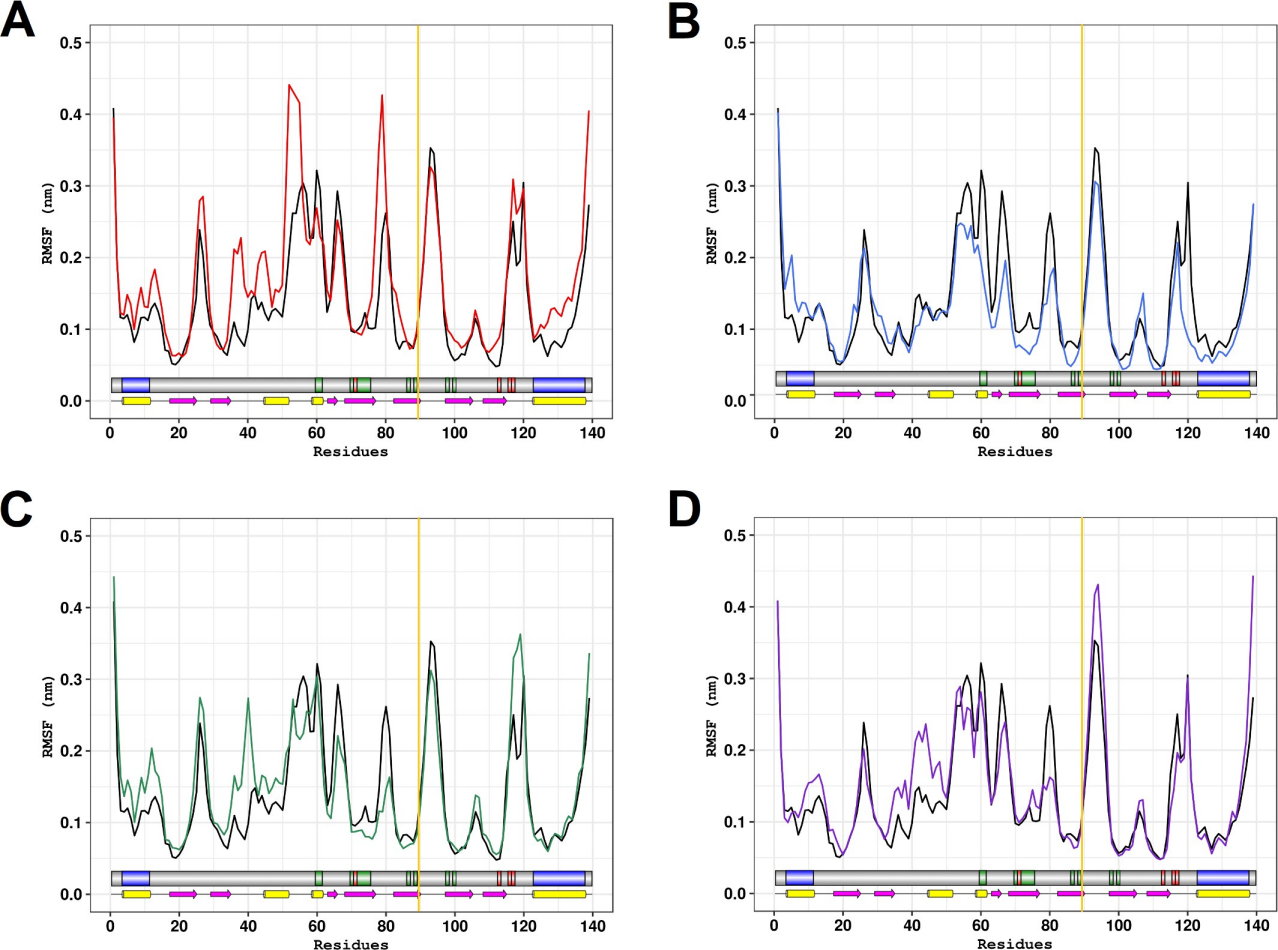
RMSF of the wild-type PFN1 and its natural variants. The RMSF of each residue of the PFN1 wild-type and variants at 300K is shown. Schematic representations of PFN1 domains and secondary structure are shown to further comparison. The PLP binding domain and actin-binding domains of PFN1 are represented in blue and green, respectively. The PFN1 mutation sites are colored red. Alpha-helices are represented by magenta arrows, beta-strands are represented by yellow barrels, and the coils are represented by the thin black lines. The dark yellow line shows the residue threonine 89. (A) The wild type is represented in black and variant C71G is represented in red. (B) The wild type is represented in black and variant M114T is represented in blue. (C) The wild type is represented in black and variant E117G is represented in green. (D) The wild type is represented in black and variant G118V is represented in purple.

The RMSD analysis, however, only provides information about the overall structure fluctuations [61]. We then performed RMSF analysis to obtain local information. RMSF is a useful parameter to describe the flexibility of protein residues throughout the simulation [4,61]. As shown in Fig 5, all studied variants presented altered flexibility in the actin-binding, PLP-binding domains and adjacent regions throughout the simulations when compared to the wild-type PFN1. However, none of the variants presented altered flexibility at the residue threonine 89.

The C71G variant presented increased flexibility at the actin-binding domain and adjacent regions, especially at the region comprised between the residues 50–56 and 75–79. It also had an increased flexibility at the N and C-terminal helices of the PLP-binding domain. In addition, this variant presented increased flexibility especially at the coils regions.

The M114T variant, in turn, presented reduced flexibility at the actin-binding domain and adjacent regions, especially at the region comprised between the residues 73–82 and 92–94. It also had increased flexibility at the N-terminal helix of the PLP-binding domain and decreased flexibility at the C-terminal helix of the PLP-binding domain. Moreover, this variant presented decreased flexibility especially at the coil and helices regions.

The E117G variant presented reduced flexibility at the actin-binding domain and adjacent regions, especially at the region comprised between the residues 64–68 and 77–81. It also had increased flexibility at the N-terminal helix of the PLP-binding domain and an increased flexibility in a region adjacent to the C-terminal helix of the PLP-binding domain (residues 116–120). In addition, this variant presented decreased flexibility especially at the coil regions, except for the region comprised between the residues 36–42, which had an increased flexibility when compared to the wild-type.

The G118V, in turn, presented decreased flexibility in adjacent regions to the actin-binding domains, except for the region comprised between the residues (93–96). It also had an increased flexibility at the N-terminal helix of the PLP-binding domain. Moreover, this variant presented decreased flexibility especially at the coil regions, except for the region comprised between the residues 36–50, which had an increased flexibility when compared to the wild-type PFN1.

Since protein flexibility has a wide influence on the thermodynamics of binding [62,63] the flexibility changes observed in the PLP,actin-binding domain and adjacent regions of PFN1 variants might be related to the known altered binding ability of these variants [16].

**Fig 6:**
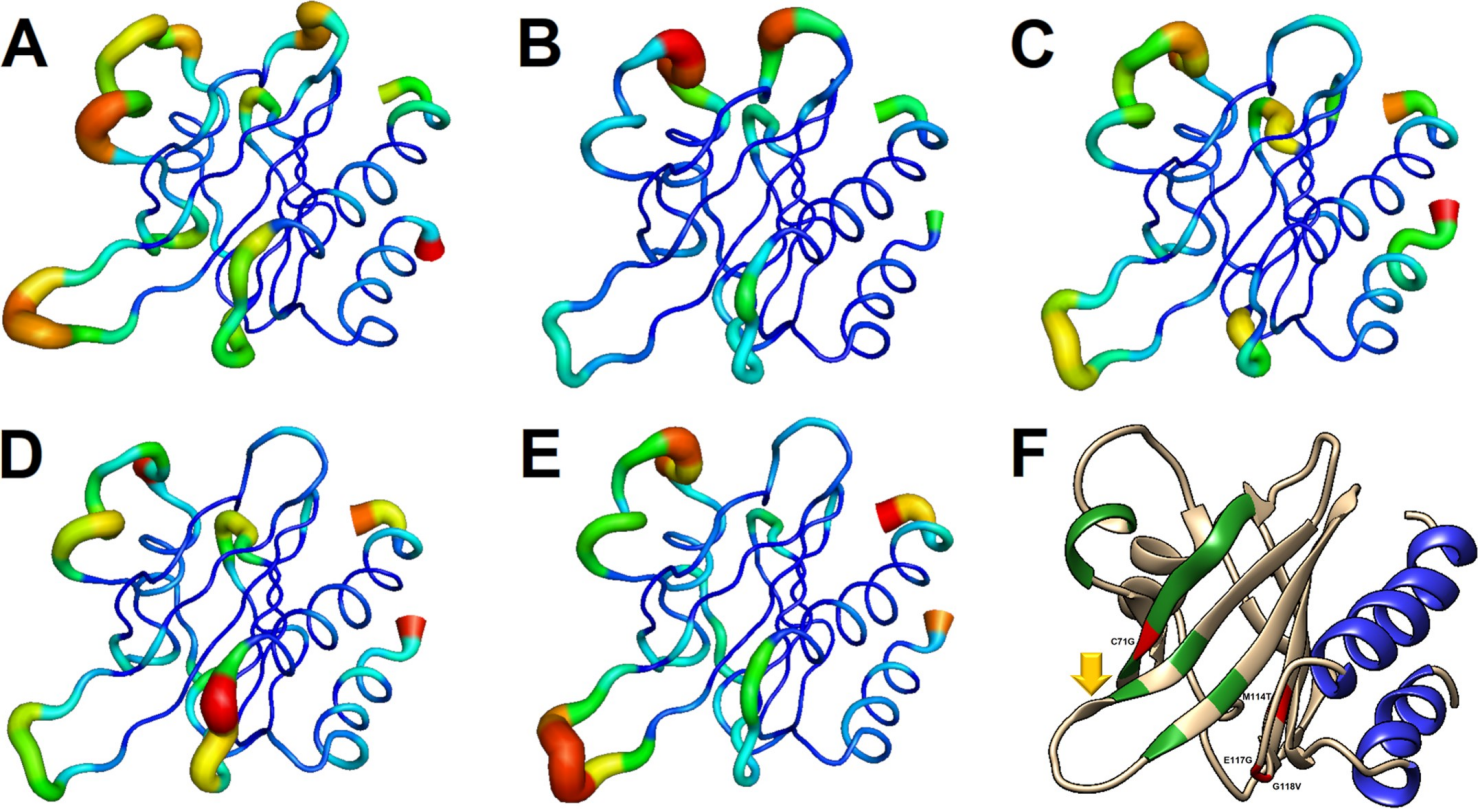
The B-factor representation of PFN1 wild-type and its natural variants. The B-factor for each residue of the PFN1 wild-type and variants represented in a coloring-thickness scheme. Red and bulky structures represent high values and dark blue and thin structures represent low values. (A) B-factor representation of the wild type PFN1. (B) B-factor representation of the C71G variant. (C) B-factor representation of the M114T variant. (D) B-factor representation of the E117G variant. (E) B-factor representation of the G118V variant. (F) Schematic representation of PFN1 structure to further comparison. The PLP binding domain and actin-binding domains of PFN1 are represented in blue and green, respectively. The PFN1 mutation sites are colored red. The dark yellow arrow shows the residue threonine 89.

The structural flexibility can also be assessed throughout the simulation by analyzing the B-factor [4]. As well as the RMSF, B-factor is useful for describing the flexibility of protein residues [4,64]. The distribution of B-factors along a protein structure is an important indicator of its dynamics [65]. We then projected the B-factor values calculated for each PFN1 residue in the protein surface (Fig 6).

The C71G variant presented increased flexibility at adjacent regions to the actin-binding domain of PFN1. The M114T variant, in turn, presented decreased flexibility at the actin-binding domain and adjacent regions, as well as increased flexibility at the PLP-binding domain and adjacent regions. The E117G variant presented decreased flexibility in adjacent regions of the actin-binding domain, as well as increased flexibility in adjacent regions of the PLP-binding domain. The G118V variant, in turn, presented decreased flexibility at the actin binding domain and adjacent regions, except for the loop that connects the fifth and sixth beta-strands, which presented increased flexibility when compared to the wild-type. In addition to reaffirming the flexibility alterations observed in the RMSF analysis, B-factor analysis also provided an interesting graphical representation of structural flexibility.

**Fig 7:**
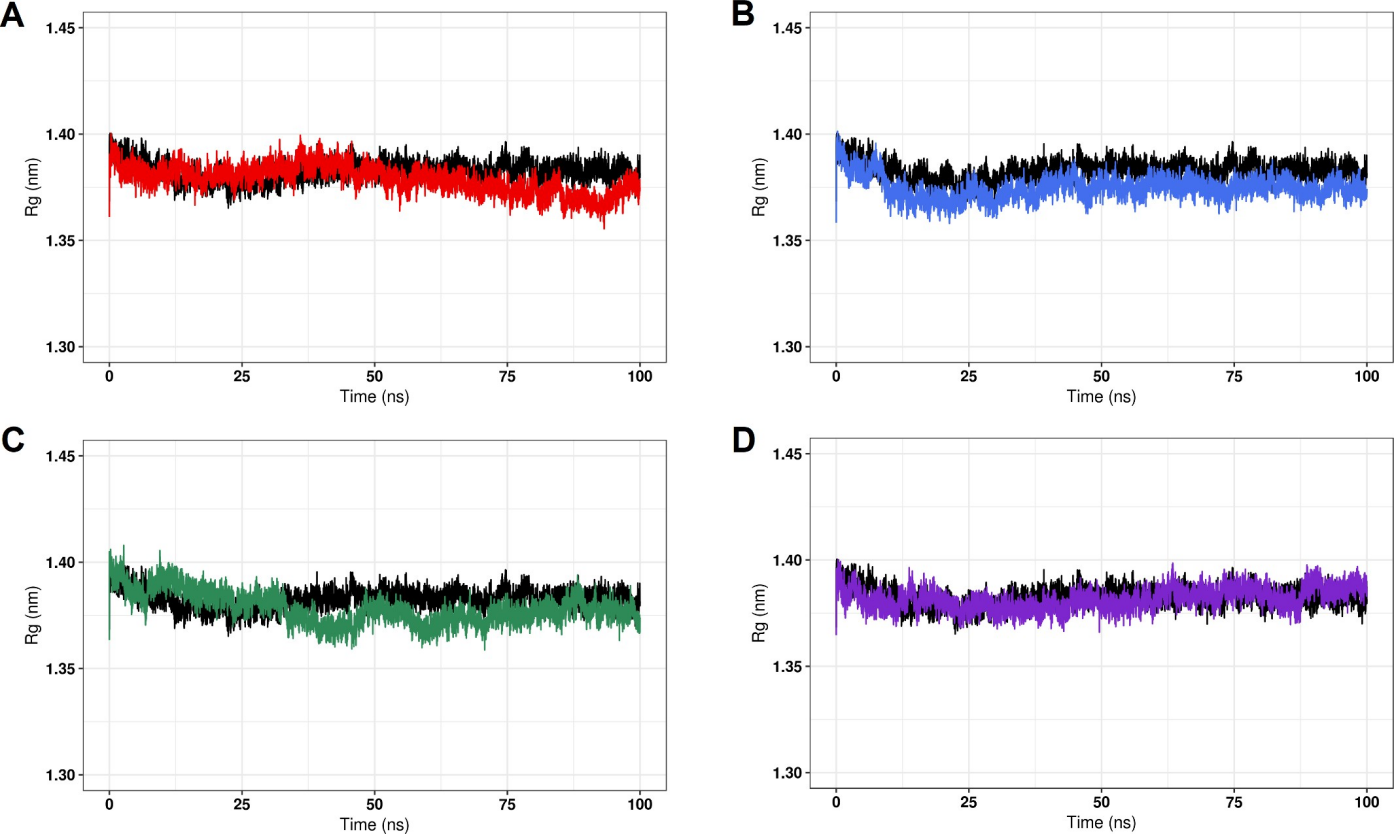
The radius of gyration (Rg) of the wild-type PFN1 and its natural variants. The Rg for the Cα atoms of the wild-type PFN1 and its natural variants at 300 K are shown as a function of time. (A) The wild type is represented in black and variant C71G is represented in red. (B) The wild type is represented in black and variant M114T is represented in blue. (C) The wild type is represented in black and variant E117G is represented in green. (D) The wild type is represented in black and variant G118V is represented in purple.

The Rg analysis is useful for describing the overall dimensions of protein structures throughout the simulation [4,52,61]. As shown in Fig 7, the average Rg value of the wild-type structure (1.383±0.02) is similar to those of the C71G (1.379±0.01nm), M114T (1.375±0.01nm), E117G (1.378±0.01nm), and G118V (1.381±0.01nm) variants. These results suggest that the C71G, M114T, E117G, and G118V variants are as compact as the wild–type PFN1

**Fig 8:**
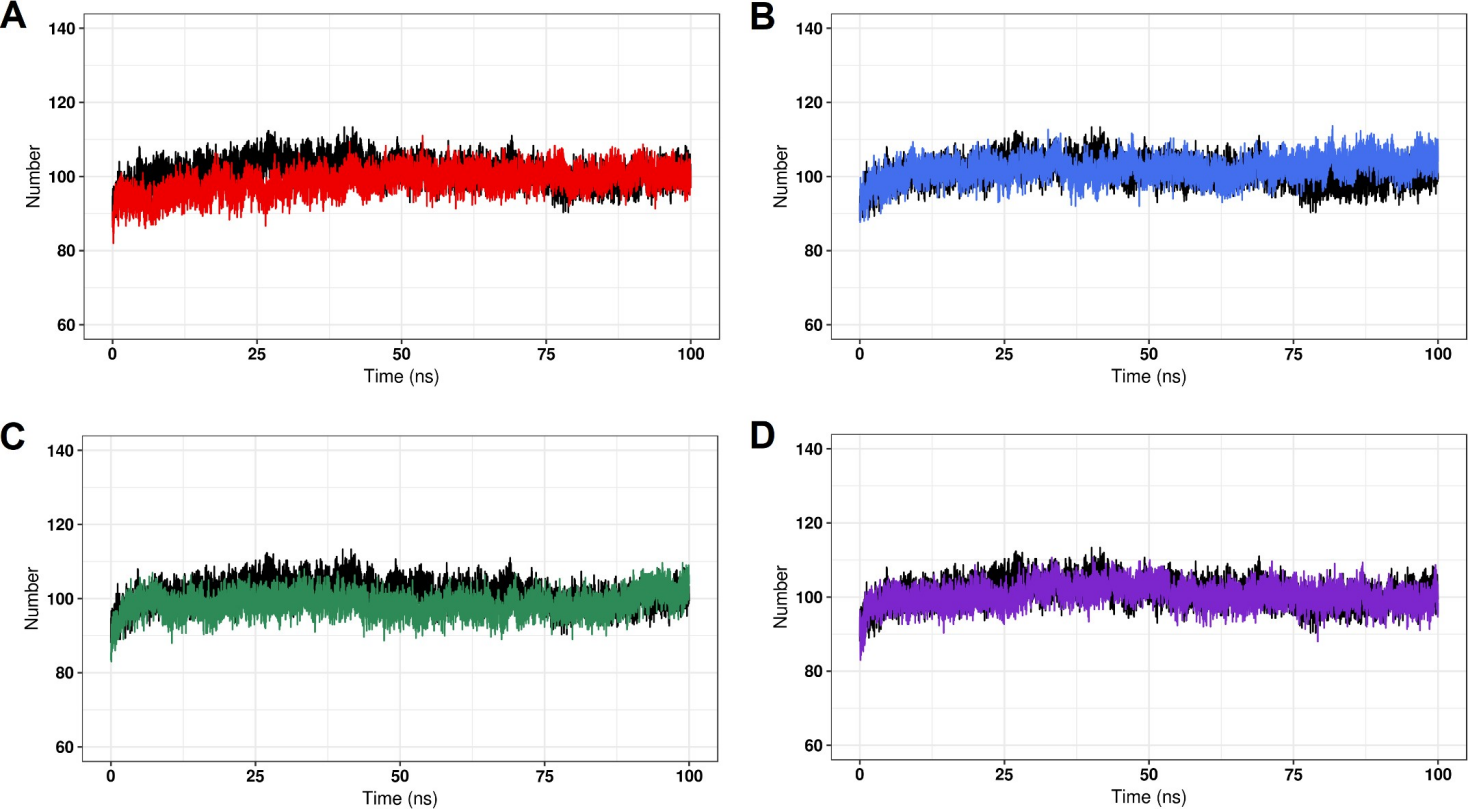
Intramolecular hydrogen bonds (Hb) for the wild-type PFN1 and its natural variants. The number of intramolecular Hb formed at 300 K throughout the simulations is shown as a function of time. (A) The wild-type is represented in black and variant C71G is represented in red. (B) The wild type is represented in black and variant M114T is represented in blue. (C) The wild type is represented in black and variant E117G is represented in green. (D) The wild type is represented in black and variant G118V is represented in purple.

The stability of protein structures can be assessed throughout the simulation by analyzing the formation of intramolecular hydrogen bonds [66]. As shown in Fig 8, the average number of intramolecular hydrogen bonds formed in the wild-type simulation (101.26±5.85) is similar to those of the C71G (98.67±6.80), M114T (102.04±5.13), E117G (98.50±5.46), and G118V (100.41±6.11) simulations. It suggests that all studied variants are as stable as the wild–type PFN1.

**Fig 9:**
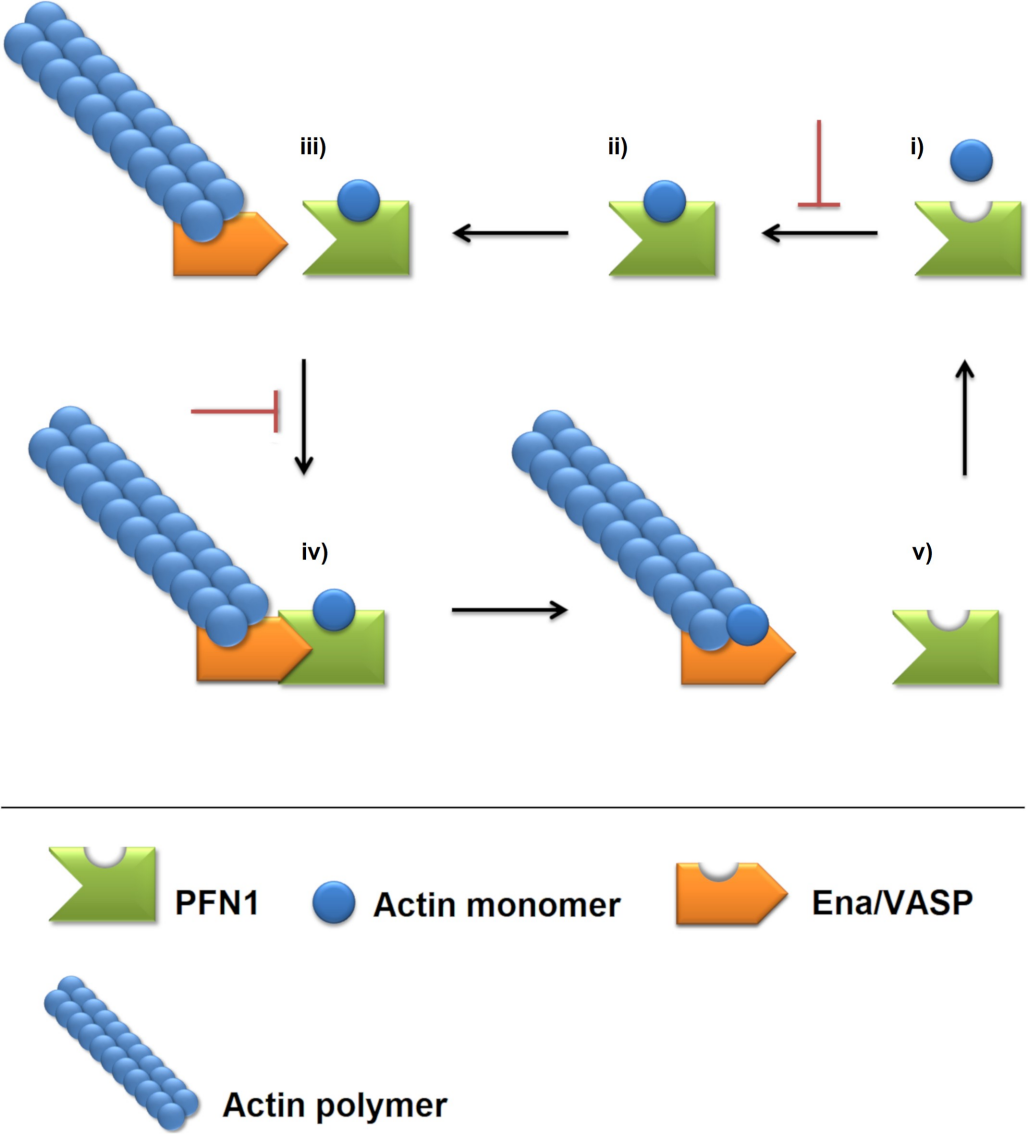
Schematic representation of the PFN1 mechanism of action and how it can be disrupted by missense mutations. PFN1 is represented in green, actin monomer is represented in blue, Ena/VASP is represented in orange, and the actin polymer is represented by the blue chained filament. Black arrows indicate the normal PFN1 mechanism of action, while the inhibitory arrow (red) indicates how this mechanism could be disrupted by missense mutations. i) The unbound PFN1 is able to interact with actin monomers. ii) PFN1 interacts through its actin-binding domain with an actin monomer. iii) Upon binding to the actin monomer, PFN1 interacts through its PLP-binding domain with Enabled/vasodilator-stimulated phosphoproteins (Ena/VASP). iv) Ena/VASP, in turn, is responsible for adding the actin monomer captured by PFN1 to the crescent actin filament polymer. v) After the delivery of actin monomer, the PFN1 is released from Ena/VASP. The C71G, M114T, E117G and G118V missense mutations in PFN1 are known to affect the actin, and PLP-binding of BDNF. We proposed that it may occur due to the flexibility alterations at the actin and PLP-binding domains and adjacent residues of PFN1.

The MD analyzes therefore suggested that the studied mutations could affect the PFN1 flexibility at the actin and PLP-binding domains, and, consequently, their intermolecular interactions. It may explain the known altered binding ability of the C71G, M114T, E117G and G118V variants [16]. Moreover, considering that the PFN1 functions are mediated by its actin and PLP-binding ability [9,12], these findings could be also related to the functional impairment of PFN1 upon C71G, M114T, E117G, and G118V mutations (Fig 9), and their involvement in ALS development [9,17].

### PFN1 database

Visualization and analysis of intricate 3D structures of macromolecules, such as proteins, are essential to provide insights into their biological processes [49]. For such purpose, there is a wide range of graphics software and web-based viewers currently available [29,67]. Amongst them, Jmol, which is a widely used open-source viewer of 3D structures [49]. However, this application is falling into disuse because its web-based version is embedded as a Java applet, a plug-in that is no longer supported on many devices and browsers due to security concerns [29,68,69]. In this scenario, JSmol, an HTML5-based equivalent of Jmol [49], comes as a great solution, because it requires no Java applets to run and produces identical graphical results [68]. We, therefore, developed a curated database of human variants using JSmol.

The PFN1 results presented in this paper are stored in SNPMOL, the human-curated database developed by our group (http://www.snpmol.org/). The database is freely available for biologists and clinicians to exploit the PFN1 variants described here and their functional and structural alterations. SNPMOL interface allows users to quickly retrieve and analyze the predicted effects and theoretical models of PFN1 variants. Understanding their effects on PFN1 structure and function may help the development of new drugs and treatments for ALS [22], as well as facilitating the design of further experiments [70].

## Conclusions

In this paper, we analyzed the effects of PFN1 nsSNVs using ten functional and stability prediction algorithms, an evolutionary algorithm, and MD simulations. The functional prediction algorithms used here showed high accuracy in detecting the known deleterious potential of the C71G, M114T, and G118V mutations, but not E117G. The functional prediction analysis also showed that it is important to use a variety of algorithms to determine the deleterious effects of mutations. The stability prediction suggested that the ALS-related mutations could destabilize PFN1. The evolutionary conservation analysis indicated that the mutations C71G, M114T, E117G, and G118V occur in highly conserved positions. The MD analyses suggested that the studied mutations could affect the PFN1 flexibility at the actin and PLP-binding domains, and consequently, their intermolecular interactions. It may be therefore related to the functional impairment of PFN1 upon C71G, M114T, E117G and G118V mutations, and their involvement in ALS development. We also developed a human-curated database, SNPMOL (http://www.snpmol.org/), containing the results presented in this paper for biologists and clinicians to exploit PFN1 and its natural variants. Furthermore, we can conclude that computational simulations are an effective approach for the study of disease-related mutations, as well as an important ally of the experimental methods.
